# Early ultra-processed foods consumption and hyperactivity/inattention in adolescence

**DOI:** 10.11606/s1518-8787.2024058005636

**Published:** 2024-10-08

**Authors:** Ricardo Campos Ferreira, Angela Helena Marin, Marcia Regina Vitolo, Paula Dal Bo Campagnolo

**Affiliations:** I Universidade do Vale do Rio dos Sinos Programa de Pós-graduação em Nutrição e Alimentos São Leopoldo RS Brasil Universidade do Vale do Rio dos Sinos. Programa de Pós-graduação em Nutrição e Alimentos. São Leopoldo, RS, Brasil; II Universidade Federal do Rio Grande do Sul Departamento de Psicologia do Desenvolvimento e da Personalidade Porto Alegre RS Brasil Universidade Federal do Rio Grande do Sul. Departamento de Psicologia do Desenvolvimento e da Personalidade. Porto Alegre,RS, Brasil; III Universidade Federal do Rio Grande do Sul Programa de Pós-graduação em Psicologia Porto Alegre RS Brasil Universidade Federal do Rio Grande do Sul. Programa de Pós-graduação em Psicologia. Porto Alegre, RS, Brasil; IV Universidade Federal de Ciências da Saúde de Porto Alegre Departamento de Nutrição Porto Alegre RS Brasil Universidade Federal de Ciências da Saúde de Porto Alegre. Departamento de Nutrição. Porto Alegre, RS, Brasil

**Keywords:** Adolescent, Longitudinal Studies, Food Additives

## Abstract

**OBJECTIVE:**

To investigate the relationship between childhood consumption of ultra-processed foods and symptoms of hyperactivity/inattention in adolescents from São Leopoldo, a city in southern Brazil.

**METHODS:**

Data were collected at four distinct stages: when participants were 12-16 months old in 2001 and 2002 and later when they were 3–4, 7–8, and 12–13 years old. During the interview at 12–16 months, mothers were asked about the introduction of sugar in their child’s diet. Two 24-hour recall surveys were conducted with children aged 3–4, 7–8, and 12–13 years to assess their consumption of ultra-processed foods. At the age of 12–13 years, the participants completed the Hyperactivity/Inattention subscale of the Strengths and Difficulties Questionnaire (SDQ), which screens for mental health problems.

**RESULTS:**

Among the 173 adolescents, 22.5% exhibited hyperactivity symptoms. The consumption of ultra-processed foods in grams, kilocalories, and as a percentage of energy intake at 3-4 years old were found to be predictors of hyperactivity/inattention symptoms (RR: 0.81, 95%CI: 0.69–0.95; RR: 1.01, 95%CI: 1.00–1.02; RR: 1.02, 95%CI:1.01–1.02; RR: 1.25, 95%CI:1.04–1.51, respectively).

**CONCLUSION:**

The consumption of ultra-processed foods at an early age was associated with hyperactivity and inattention symptoms in adolescence.

## INTRODUCTION

Attention Deficit Hyperactivity Disorder (ADHD) is a neurodevelopmental disorder characterized by severe inattention, hyperactivity, and impulsivity, lasting from childhood to adulthood in most cases^1^. A systematic review on the prevalence of mental disorders in children and adolescents identified a worldwide prevalence of 6.5% for anxiety disorders, 2.6% for depressive disorder, and 3.5% for ADHD^2^. Understanding the mechanisms that trigger this neurological disorder is essential for its adequate control.

The complex etiology of ADHD involves genetic, neurological, and environmental factors, among which unhealthy diets or nutrient deficiencies have received attention around the world^3;4^. Products with high sugar content reduce dopaminergic responses, resulting in cortical inhibition directly related to ADHD. The chronic effects of excessive sugar intake can lead to changes in mesolimbic dopamine signaling, which may contribute to symptoms associated with ADHD^5^.

Diets based on ultra-processed foods have higher energy density and contain excessive amounts of fat, sugar, sodium, and other substances such as dyes, flavorings, and flavor enhancers^6^. There is a legitimate concern in terms of public health because the consumption of this diet by preschoolers is already higher than 40% of the total energy value of a diet^8^. Thus, considering the characteristics of ultra-processed products and the studies mentioned above^5^, which indicated an association between the consumption of saturated fat and refined sugar and the risk of hyperactivity or ADHD occurrence, this study aimed to verify whether there is an association between the consumption of ultra-processed foods in preschool age and symptoms of hyperactivity in adolescence.

## METHODS

This study used data from children who participated in a randomized trial of dietary counseling on breastfeeding and dietary practices during the first year of life^9^. The children were born at a hospital that serves low-income populations in the city of São Leopoldo, between October 2001 and July 2002. The inclusion criteria were full-term (> 37 weeks) babies with a birth weight > 2,500 g. The exclusion criteria were HIV-positive mothers, congenital malformations, and children admitted to the intensive care unit.

The study comprised four phases of data collection, through home interviews with mothers/children: the first when the children were 12–16 months old (n = 397 children), the second when they were 3–4 years old (n = 354), the third when the children were 7–8 years old (n = 315), and the fourth when they were adolescents aged 12–13 years (n = 211). The analyses of the present study are longitudinal because the intervention in the first year of life had no impact on the outcomes studied. Because literature data are scarce, the sample size in the present study was calculated using WinPEPI software, version 11.43, through a pilot study with 45 adolescents. Considering a significance level of 5% and power of 85%, a prevalence of hyperactivity of 25%, and a minimum effect size of 0.6 standard deviations between groups, a minimum sample size of 155 adolescents was determined.

The child’s sex, birth weight, and gestational age were obtained from hospital records. The following demographic and maternal information was assessed when the children reached an age of 12–16 months by face-to-face interviews with mothers: maternal schooling, maternal weight and height, maternal age at child’s birth, maternal smoking in pregnancy, and child skin color.

### Dietary Data

During the 12- to 16-month interview, mothers were asked about the month in which sugar was introduced into the child’s diet. At 3–4 and 7–8 years of age, two 24-hour dietary recalls were performed for each child on non-consecutive days, and the average of the values obtained was used. For children aged 3–4 years, mothers or other caregivers provided the recall information. For children aged 7–8 years, recalls were self-reported with the assistance of mothers or other caregivers. To quantify food portion size, pictures were used to illustrate standard household measurements, such as teaspoons, tablespoons, and cups. Dietary information was entered in NutWin software (version 1.5; Federal University of São Paulo) to determine the total energy of the diet. The children’s diet was assessed using the NOVA food classification system proposed by Monteiro et al.^10^. NOVA classifies all foods according to the nature, extent, and purpose of the processing they undergo into four groups: unprocessed and minimally processed foods, including foods that are fresh or processed without the addition of substances such as salt, sugar, oils, or fats and infrequently contain additives (Group 1); processed culinary ingredients, designed to be combined with foods to make meals and dishes (Group 2); processed foods (Group 3), relatively simple products made by adding sugar, oil, salt, or other Group 2 substances to Group 1 foods; and ultra-processed foods and drink products (Group 4), which are formulations manufactured using several ingredients and a series of processes and typically including little or no fresh food (such as soft drinks, sweet or savory packaged snacks, processed meats, and pre-prepared frozen dishes). For the purposes of this study, only Group 4 was analyzed. The quantity (grams) of each food and the total energy value were determined, and the average of the two 24-hour recall surveys was used in the analyses. The consumption of ultra-processed products was estimated using the Multiple Source Method^11^(MSM; https://msm.dife.de/) to correct for intra- and inter-individual variability in dietary data.

### Anthropometric Measurements

At the age of 12–16 months, weight was measured using a portable digital scale (Techline, São Paulo, Brazil), and length was measured using an infant stadiometer (Serwital Inc, Porto Alegre, Brazil). At the age of 3–4, 7–8, and 12–13 years, children were weighed in light clothing without shoes on a digital scale (Techline, São Paulo, Brazil), and the standing height was measured to the nearest 0.1 cm using a stadiometer (SECA, Hamburg, Germany). All measures were converted into z-scores of BMI-for-age based on the World Health Organization Growth Standards, and z-scores higher than +1 indicated a risk of overweight^12, 13^.

### Strengths and Difficulties Questionnaire Applied to 12–13-Year-Old Adolescents

The Strengths and Difficulties Questionnaire (SDQ), an adolescent version developed by Godman^14^ and validated in Brazil by Fleitlich et al.^15^, was used in this study. The instrument, which is a mental health screening tool, was answered by the participants themselves (version intended for adolescents aged 11–16 years). It asks about 25 items, divided between 5 subscales: prosocial behavior, hyperactivity, and emotional, conduct, and peer relationship problems, with 5 items in each scale. The answers can be false, approximately true or true, and each item receives a specific score. The sum of each scale and the total sum allows the classification of adolescents into three categories: normal, borderline, or abnormal development^14^. In this study, only the hyperactivity/inattention subscale was used. On this subscale, a higher score indicates a greater number of complaints. Adolescents classified as borderline or abnormal were considered to have hyperactivity/inattention symptoms.

### Statistical Analysis

Variables were described using mean and standard deviation (normally distributed data) or median and interquartile range (non-normally distributed data) and percentage frequency. The Mann–Whitney test was used to assess differences in dietary variables between the groups with or without hyperactivity/inattention symptoms. Unadjusted and adjusted relative risks of hyperactivity/inattention symptoms were estimated using Poisson regression analysis with robust variance. First, the relative risk and 95% CI of each sociodemographic, maternal, and anthropometric variable were separately estimated. Then, multivariate modeling was performed between dietary variables and hyperactivity/inattention symptoms using sociodemographic, maternal, and anthropometric variables as confounders. For the Multivariate analysis, the grams, kcal, and percentage of energy from ultra-processed food at 3–4 and 7–8 years were divided by 10 to evaluate the effect of an increase of 10 grams, 10 kcal, or 10% of ultra-processed food on hyperactivity/inattention symptoms in adolescents. All statistical analyses were performed using SPSS version 19.0 and the significance level considered was p < 0.05.

The study protocol was approved in September 2013 by the Ethics Committee of Universidade do Vale do Rio dos Sinos (number 18426813.4.0000.5344. Interviews were conducted after the mother or guardian agreed to participate in the study and signed the Free and Informed Consent Form.

## RESULTS

Among the 500 children recruited at birth, 397 were evaluated at 12–16 months, 345 at 3–4 years, 315 at 7–8 years, and 214 at 12–13 years of age. No differences were observed between individuals who left or continued in the study in relation to family income (p = 0.648). A total of 173 12–13 years old adolescents completed the Strengths and Difficulties Questionnaire. The sample analyzed in the present study comprised 39.9% girls, and half of the mothers (51.4%) had less than 8 years of schooling. Hyperactivity symptoms were noted in 39 participants (22.5%). The univariate analysis showed no association between sociodemographic factors, maternal factors, and nutritional status and hyperactivity/inattention symptoms in adolescents (Table 1). Dietary analysis showed higher energy intake from ultra-processed foods at 3–4 years of age among adolescents with hyperactivity/inattention symptoms (p=0.006) (Table 2). In the multivariate analysis, using sociodemographic, maternal, and anthropometric variables as possible confounders, the consumption of ultra-processed foods in grams, kilocalories, and percentage of energy intake at 3–4 years were shown to be predictors of hyperactivity/inattention symptoms (RR: 1.01, 95% CI: 1.00–1.02; RR: 1.02, 95% CI:1.01–1.02; RR: 1.25, 95% CI:1.04–1.51, respectively; Table 3).

**Table 1:** Relationship between hyperactivity/inattention symptoms and sociodemographic factors, maternal factors, and nutritional status.

Variable	Hyperactivity/ Inattention - n (%)	RR (95%CI)	p-value
Sex			
Boys	21 (20.2)	0.78 (0.45–1.36)	0.391
Girls	18 (26.1)	1	
Skin color			
White	16 (23.5)	1.01 (0.58–1.78)	0.956
Non-white	22 (23.2)	1	
Maternal schooling (years)			
≥ 8	24 (28.6)	1.13 (1.00–1.27)	0.059
< 8	15 (16.9)	1	
Maternal BMI (kg/m^2^)			
> 30	22 (25.6)	1.12 (0.58–2.13)	0.737
≤ 30	15 (20.5)	1	
Maternal smoking during pregnancy			
Yes	4 (22.4)	0.99 (0.40–2.48)	0.988
No	32 (21.1)	1	
Gestational age (weeks)			
< 40	19 (23.8)	1.02 (0.90–1.15)	0.783
≥ 40	20 (22.0)	1	
Maternal age at childbirth (years)			
< 20	7 (26.9)	1.05 (0.88–1.27)	0.570
≥ 20	32 (21.6)	1	
Child BMI at 12 months (z-score)			
> +1	16 (25.0)	1.23 (0.52–2.90)	0.635
≤ +1	23 (21.5)	1	
Child BMI at 4 years of age (z-score)			
> +1	8 (23.5)	1.01 (0.51–1.99)	0.978
≤ +1	31 (23.3)	1	
Child BMI at 8 years of age (z-score)			
> +1	13 (27.7)	1.38 (0.77–2.47)	0.273
≤ +1	25 (20.0)	1	

BMI: body mass index.

**Table 2:** Association between hyperactivity/inattention symptoms in adolescents and the consumption of ultra-processed foods.

Variable	Hyperactivity/ inattention	No hyperactivity/ inattention	p-value^a^
	
	Median (IQR)	Median (IQR)	
At 12–16 months			
Sugar in the child’s diet (mo)	3.0 (0.5–6.0)	3.0 (5.0)	0.703
At 3–4 years			
Ultra-processed products (g)	341.8 (200.1–411.7)	296.3 (167.2–419.9)	0.306
Ultra-processed products (kcal)	786.6 (527.9–966.2)	552.6 (374.3–846.6)	0.006
% energy from ultra-processed products	46.3 (36.2–54.8)	40.9 (29.7–50.9)	0.084
At 7–8 years			
Ultra-processed products (g)	306.3 (198.0–480.6)	356.0 (233.4–526.1)	0.218
Ultra-processed products (kcal)	718.3 (559.9–918.9)	773.1 (533.91053.7)	0.325
% energy from ultra-processed products	44.7 (35.5–55.3)	49.9 (40.7–60.1)	0.071

IQR: interquartile range; TEV: total energetic value.

^a^ Mann-Whitney test.

**Table 3:** Poisson regression of ultra-processed food consumption in early childhood and hyperactivity/inattention symptoms in adolescence.

Variable	RR (95% CI)	p-value
At 12–16 months		
Introduction of sugar into the child’s diet (mo)^a^	0.97 (0.87–1.08)	0.612
At 3–4 years		
grams of ultra-processed products^b^	1.01 (1.00–1.02)	0.011
kcal for ultra-processed products^b^	1.02 (1.01–1.02)	< 0.001
% energy from ultra-processed products^b^	1.25 (1.04–1.51)	0.018
At 7–8 years		
grams of ultra-processed products	0.99 (0.97–1.00)	0.056
kcal from ultra-processed products^c^	0.99 (0.99–1.00)	0.263
% energy from ultra-processed products	0.85 (0.72–1.01)	0.064

RR: Relative risk; 95% CI: 95% confidence interval; mo: months; TEV: Total energy value.

^a^Adjusted for: sex, skin color, gestational age, maternal smoking during pregnancy, maternal schooling, maternal obesity, maternal age, and child overweight at 12 months of age.

^b^Adjusted for: sex, skin color, gestational age, maternal smoking during pregnancy, maternal schooling, maternal obesity, maternal age, and child overweight at 4 years of age.

^c^Adjusted for: sex, skin color, gestational age, maternal smoking during pregnancy, maternal schooling, maternal obesity, maternal age, and child overweight at 8 years of age.

## DISCUSSION

This study identified ultra-processed food intake at the age of 3–4 years as a predictor of hyperactivity/inattention symptoms in adolescents. From the nutritional point of view, some hypotheses can explain these results. Sugars, artificial dyes, and chemical preservatives, which are found in ultra-processed foods, are associated with an increased risk of ADHD^16^. In addition to sugars and additives, the quality of diet has been studied to identify possible relationships with hyperactivity in children and adolescents.

A case-control study showed that children with ADHD have different dietary patterns; thus, dietary and nutritional factors may play a role in the pathophysiology of ADHD. A higher intake of refined grains and lower intake of dairy products, calcium, and vitamin B-2 were observed in children with ADHD compared with those in the control group^17^. Similarly, a recent meta-analysis suggested that an unhealthy diet characterized by the consumption of saturated fat and refined sugar may increase the risk of ADHD, whereas a healthy diet containing fruits, vegetables, and whole grains had a protective effect against hyperactivity^7^. Data from the ALSPAC study showed that children eating a diet high in “junk food” at the age of 4 years were more likely to be in the highest tertile on the SDQ hyperactivity subscale at the age of 7 years. The association was modest but significant^18^.

Ultra-processed foods are also high in saturated fat and low in omega-3 fatty acids. Different studies have identified omega-3 as a crucial factor in the etiology of ADHD in children. There is also evidence that a diet rich in fiber, folate, and omega-3 fatty acids acts as a protective factor against the development of ADHD^19^. Studies on omega-3 go beyond the association with the development of hyperactivity and evaluate its effect on the treatment of ADHD. A systematic review showed that a diet containing omega-3 fatty acids improves the total symptoms of ADHD and that youngsters with ADHD have lower levels of DHA, EPA, n-3 PUFAs, and AA^20^. The association between ultra-processed foods and hyperactivity symptoms can be mediated by the intestinal microbiota. Current evidence shows that chronic and excessive intake of ultra-processed foods influences the function of the intestinal microbiota, with neurological effects that seem to involve mechanisms such as dysbiosis and the development of neuroinflammation^21^.

Although much remains to be understood about the role of the intestinal microbiota in hyperactivity, evidence from preliminary studies in humans suggests that components of the diet that modulate the intestinal microbiota may also influence the development or symptoms of ADHD^22^. To preserve brain health, it is important to promote adequate gut microbiota communities to prevent dysfunction of the gut-brain axis^4^. There is still little understanding of the long-term programing effects of the interaction between the intestinal microbiota and brain development during childhood. However, this is a critical area of study because disorders of the developing intestinal microbiota in early life can affect neurodevelopment and, consequently, negatively impact lifelong mental health^23^.

In the present study, it was noteworthy that consumption of ultra-processed foods at 3–4 years of age was associated with hyperactivity symptoms, which did not occur with consumption of these foods at 7–8 years of age. These findings may reflect the critical period of cognitive development during childhood. The first years of life are characterized by rapid brain development, which is fundamental for cognitive development. Our results reinforce the concept of a window of opportunity during which nutrients can affect postnatal neural development^24^.

Some specific limitations of this study should be discussed. Only children with low socioeconomic status were included, which limits the generalization of the results to other strata. In addition, only hyperactivity/inattention symptoms were evaluated, and not all the official criteria necessary to define ADHD. However, the Strengths and Difficulties Questionnaire is widely used as an international standardized instrument for measuring child behavior, and the subscale is associated with ADHD^25^. Additionally, self-reporting on the SDQ is valid for detecting emotional and behavioral problems in adolescents. Despite these limitations, the importance and relevance of this study are emphasized by the results obtained through a longitudinal design, which calls attention to the harmful effects of the consumption of ultra-processed products. These effects go beyond those already evidenced in the literature and suggest new hypotheses for future research with patients diagnosed with ADHD. The important impact of hyperactivity on biopsychosocial development, especially in childhood and adolescence, makes early changes in lifestyle extremely relevant to minimize this pathology.

The confirmation of results in other populations is important for understanding the multifactorial etiology of ADHD, as well as for the development and scale implementation of public health strategies aimed at reducing their impact by minimizing the consumption of ultra-processed products in early life. The results of this study also reinforce the importance of public policies that lead to decreased consumption of ultra-processed foods in all age groups, especially in children.

## CONCLUSIONS

The findings of this study reveal a significant association between the early consumption of ultra-processed foods and the incidence of hyperactivity symptoms among adolescents. These results underscore the urgency for heightened discourse surrounding the impact of unhealthy dietary patterns established in childhood, alongside concerns about obesity and metabolic complications. Although evidence remains relatively recent, the detrimental effects of ultra-processed foods on the psychological well-being of adolescents warrant serious consideration. Further prospective research is necessary to enhance the depth of evidence in this area.
