# ERRATUM

**DOI:** 10.11606/s1518-8787.2024058005636err

**Published:** 2024-11-27

In the article “Early ultra-processed foods consumption and hyperactivity/inattention in adolescence”, DOI: https://doi.org/10.11606/s1518-8787.2024058005636, published on the Revista de Saúde Pública.2021;58:46, RSP corrects:

**Abstract (page 1)**

Where it reads:

(RR: 0.81, 95%CI: 0.69–0.95; RR: 1.01, 95%CI: 1.00–1.02; RR: 1.02, 95%CI:1.01–1.02; RR: 1.25,

95%CI:1.04–1.51, respectively).

It should read:

(RR: 1.01, 95%CI: 1.00–1.02; RR: 1.02, 95%CI:1.01–1.02; RR: 1.25, 95%CI:1.04–1.51, respectively).

**In the line 11 of last paragraph of the Results section (page 5):**

Where it reads:

In the multivariate analysis, using sociodemographic, maternal, and anthropometric variables as possible confounders, the consumption of ultra-processed foods in grams, kilocalories, and percentage of energy intake at 3–4 years were shown to be predictors of hyperactivity/inattention symptoms (RR: 1.01, 95% CI: 1.00–1.02; RR: 1.02, 95% CI:1.01–1.02; RR: 1.25, 95% CI:1.04–1.51 (RR values refer to the effect of increasing 10 g, 10 kcal and 10%, respectively; Table 3).

**Table 3:** Poisson regression of ultra-processed food consumption in early childhood and hyperactivity/inattention symptoms in adolescence.

Variable	RR (95% CI)	p-value
At 12–16 months		
Introduction of sugar into the child’s diet (mo)^a^	0.97 (0.87–1.08)	0.612
At 3–4 years		
grams of ultra-processed products (10g)^b,d^	1.01 (1.00–1.02)	0.011
kcal for ultra-processed products (10kcal)^b,d^	1.02 (1.01–1.02)	< 0.001
% energy from ultra-processed products (10%)^b,d^	1.25 (1.04–1.51)	0.018
At 7–8 years		
grams of ultra-processed products (10g)^c,d^	0.99 (0.97–1.00)	0.056
kcal from ultra-processed products (10kcal)^c,d^	0.99 (0.99–1.00)	0.263
% energy from ultra-processed products (10%)^c,d^	0.85 (0.72–1.01)	0.064

RR: Relative risk; 95% CI: 95% confidence interval; mo: months.

^a^ Adjusted for: sex, skin color, gestational age, maternal smoking during pregnancy, maternal schooling, maternal obesity, maternal age, and child overweight at 12 months of age.

^b^ Adjusted for: sex, skin color, gestational age, maternal smoking during pregnancy, maternal schooling, maternal obesity, maternal age, and child overweight at 4 years of age.

^c^ Adjusted for: sex, skin color, gestational age, maternal smoking during pregnancy, maternal schooling, maternal obesity, maternal age, and child overweight at 8 years of age.

^d^ Values were divided by 10 to evaluate the effect of an increase of 10 grams, 10 kcal, or 10% of ultra-processed food on outcome.

It should read:

In the multivariate analysis, using sociodemographic, maternal, and anthropometric variables as possible confounders, the consumption of ultra-processed foods in grams, kilocalories, and percentage of energy intake at 3–4 years were shown to be predictors of hyperactivity/inattention symptoms: RR: 1.01, 95% CI: 1.00–1.02; RR: 1.02, 95% CI:1.01–1.02; RR: 1.25, 95% CI:1.04–1.51 (RR values refer to the effect of increasing 10 g, 10 kcal and 10%, respectively; Table 3).

**Table 3 (page 5)**

Where it reads:

**Table 3:** Poisson regression of ultra-processed food consumption in early childhood and hyperactivity/inattention symptoms in adolescence.

Variable	RR (95% CI)	p-value
At 12–16 months		
Introduction of sugar into the child’s diet (mo)^a^	0.97 (0.87–1.08)	0.612
At 3–4 years		
grams of ultra-processed products^b^	1.01 (1.00–1.02)	0.011
kcal for ultra-processed products^b^	1.02 (1.01–1.02)	< 0.001
% energy from ultra-processed products^b^	1.25 (1.04–1.51)	0.018
At 7–8 years		
grams of ultra-processed products	0.99 (0.97–1.00)	0.056
kcal from ultra-processed products^c^	0.99 (0.99–1.00)	0.263
% energy from ultra-processed products	0.85 (0.72–1.01)	0.064

RR: Relative risk; 95% CI: 95% confidence interval; mo: months; TEV: Total energy value.

^a^Adjusted for: sex, skin color, gestational age, maternal smoking during pregnancy, maternal schooling, maternal obesity, maternal age, and child overweight at 12 months of age.

^b^Adjusted for: sex, skin color, gestational age, maternal smoking during pregnancy, maternal schooling, maternal obesity, maternal age, and child overweight at 4 years of age.

^c^Adjusted for: sex, skin color, gestational age, maternal smoking during pregnancy, maternal schooling, maternal obesity, maternal age, and child overweight at 8 years of age.

It should read:

